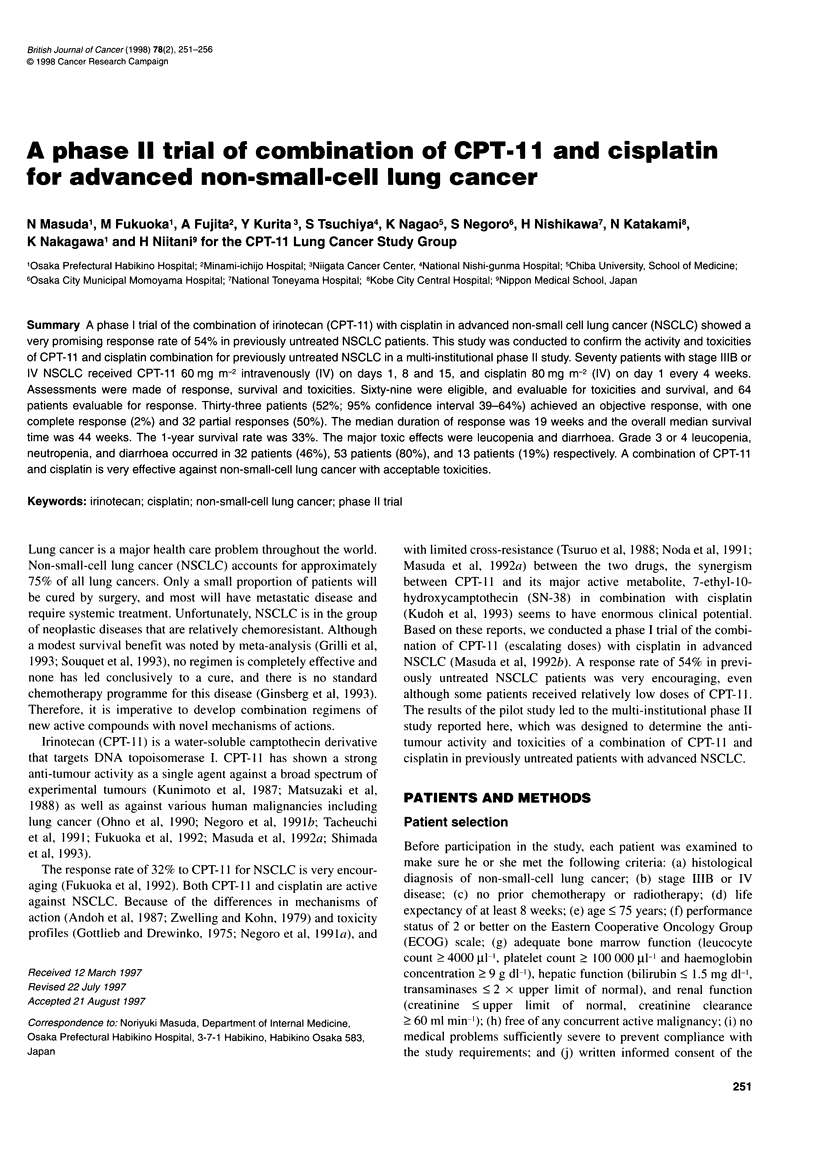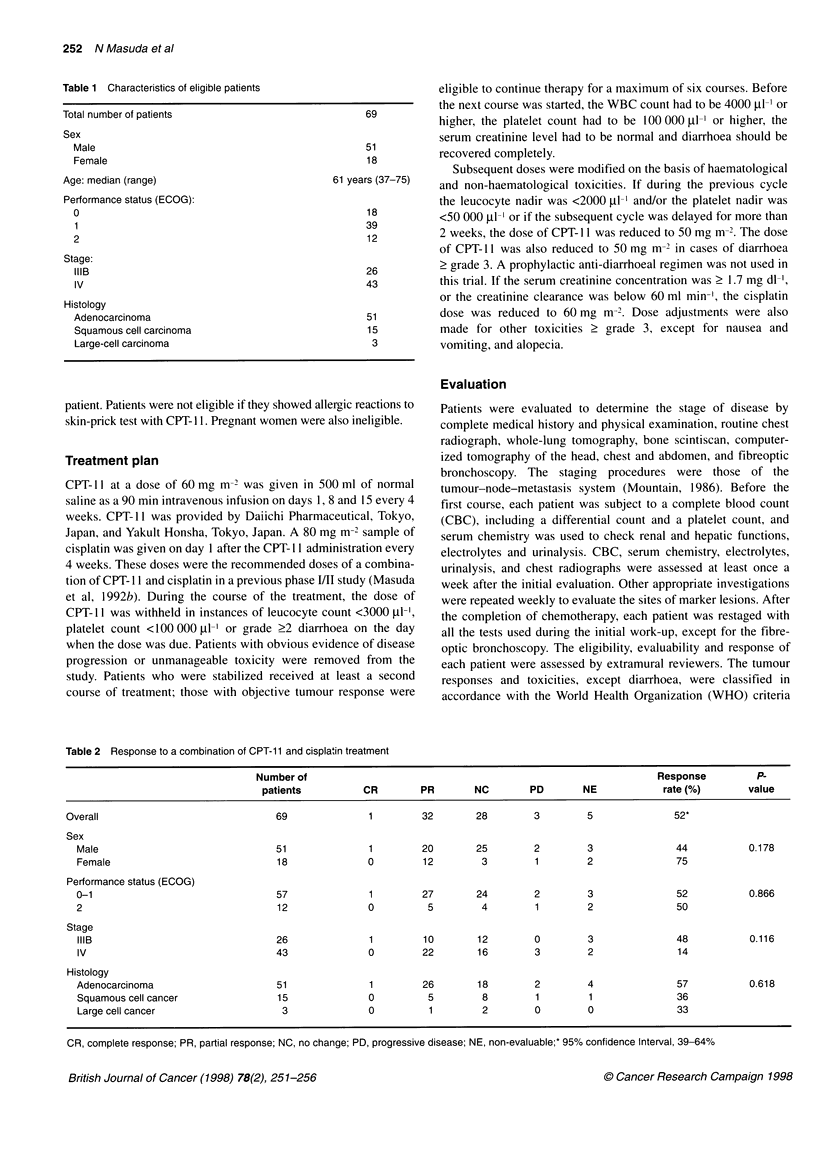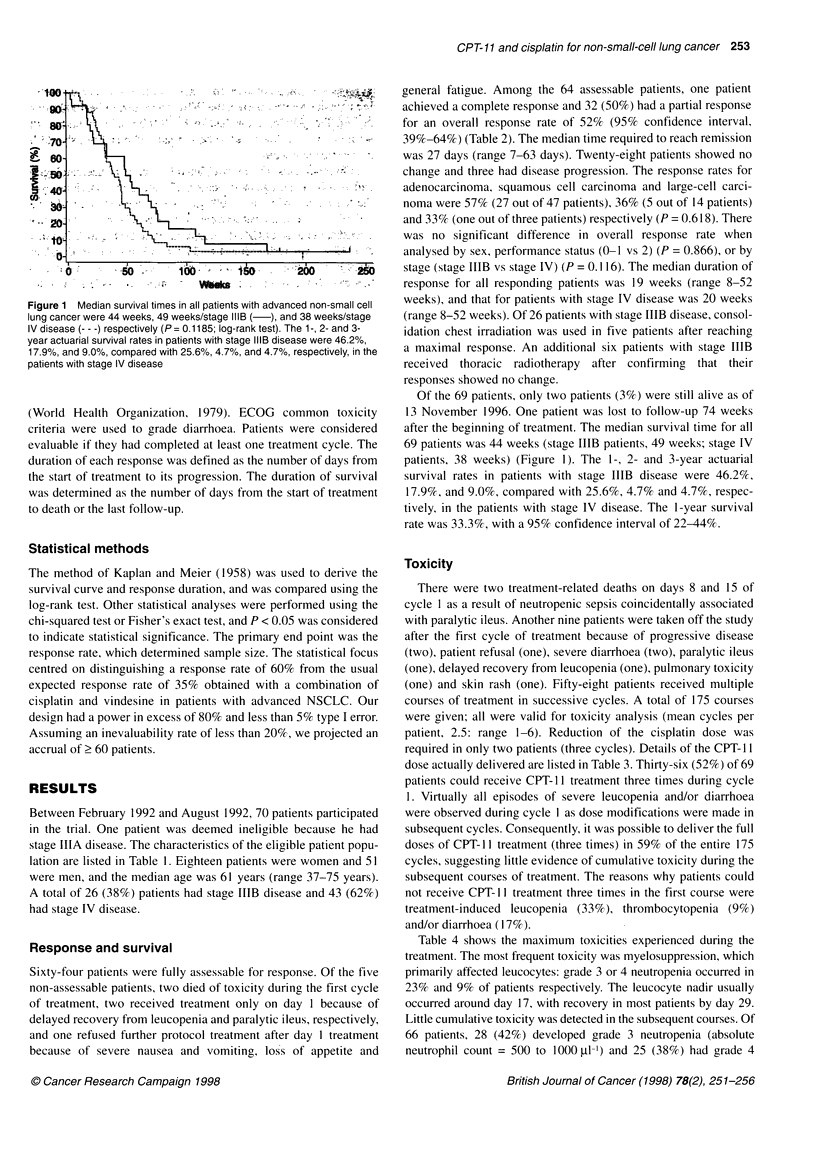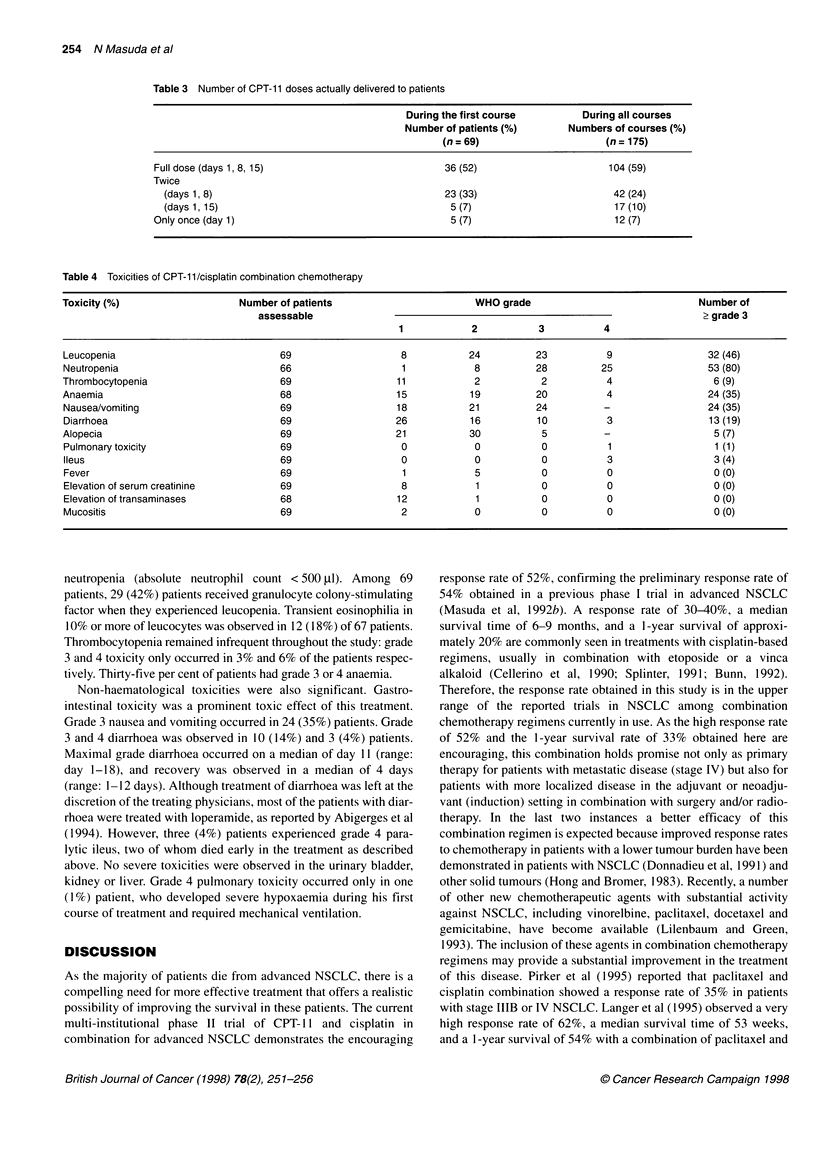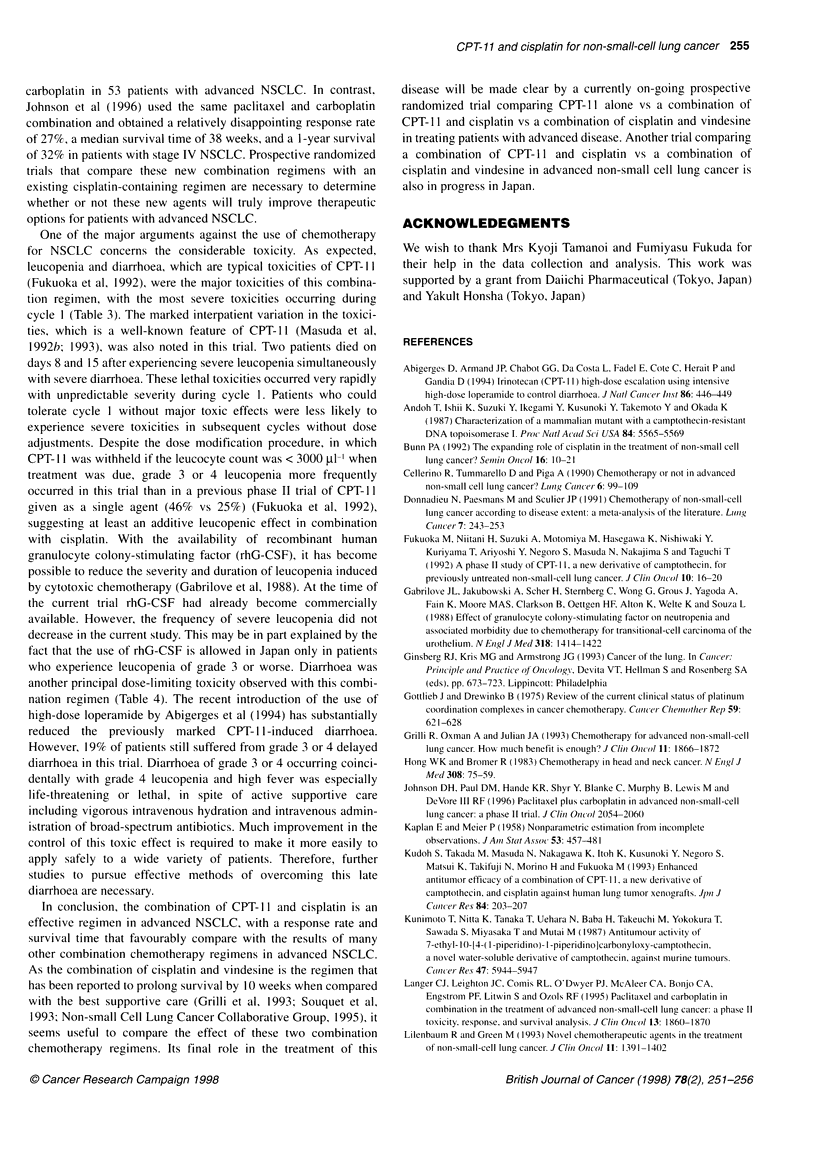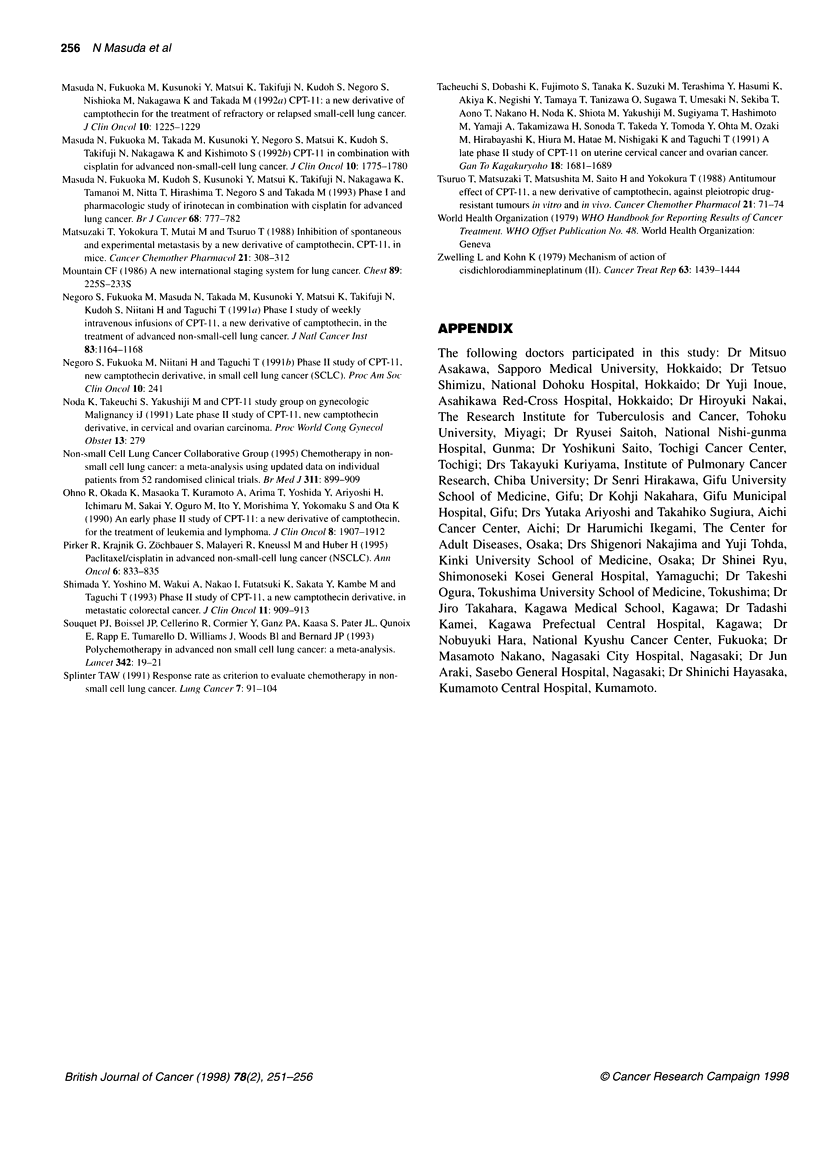# A phase II trial of combination of CPT-11 and cisplatin for advanced non-small-cell lung cancer. CPT-11 Lung Cancer Study Group.

**DOI:** 10.1038/bjc.1998.473

**Published:** 1998-07

**Authors:** N. Masuda, M. Fukuoka, A. Fujita, Y. Kurita, S. Tsuchiya, K. Nagao, S. Negoro, H. Nishikawa, N. Katakami, K. Nakagawa, H. Niitani

**Affiliations:** Osaka Prefectural Habikino Hospital, Japan.

## Abstract

A phase I trial of the combination of irinotecan (CPT-11) with cisplatin in advanced non-small cell lung cancer (NSCLC) showed a very promising response rate of 54% in previously untreated NSCLC patients. This study was conducted to confirm the activity and toxicities of CPT-11 and cisplatin combination for previously untreated NSCLC in a multi-institutional phase II study. Seventy patients with stage IIIB or IV NSCLC received CPT-11 60 mg m(-2) intravenously (i.v.) on days 1, 8 and 15, and cisplatin 80 mg m(-2) (i.v.) on day 1 every 4 weeks. Assessments were made of response, survival and toxicities. Sixty-nine were eligible, and evaluable for toxicities and survival, and 64 patients evaluable for response. Thirty-three patients (52%; 95% confidence interval 39-64%) achieved an objective response, with one complete response (2%) and 32 partial responses (50%). The median duration of response was 19 weeks and the overall median survival time was 44 weeks. The 1-year survival rate was 33%. The major toxic effects were leucopenia and diarrhoea. Grade 3 or 4 leucopenia, neutropenia, and diarrhoea occurred in 32 patients (46%), 53 patients (80%), and 13 patients (19%) respectively. A combination of CPT-11 and cisplatin is very effective against non-small-cell lung cancer with acceptable toxicities.


British Journal of Cancer (1998) 78(2), 251–256
© 1998 Cancer Research Campaign

### A phase II trial of combination of CPT-11 and cisplatin for advanced non-small-cell lung cancer

**N Masuda[1], M Fukuoka[1], A Fujita[2], Y Kurita[3], S Tsuchiya[4], K Nagao[5], S Negoro[6], H Nishikawa[7], N Katakami[8], K Nakagawa[1] and H Niitani[9] for the CPT-11 Lung Cancer Study Group**

[1]Osaka Prefectural Habikino Hospital; [2]Minami-ichijo Hospital; [3]Niigata Cancer Center, [4]National Nishi-gunma Hospital; [5]Chiba University, School of Medicine; [6]Osaka City Municipal Momoyama Hospital; [7]National Toneyama Hospital; [8]Kobe City Central Hospital; [9]Nippon Medical School, Japan

**Summary** A phase I trial of the combination of irinotecan (CPT-11) with cisplatin in advanced non-small cell lung cancer (NSCLC) showed a very promising response rate of 54% in previously untreated NSCLC patients. This study was conducted to confirm the activity and toxicities of CPT-11 and cisplatin combination for previously untreated NSCLC in a multi-institutional phase II study. Seventy patients with stage IIIB or IV NSCLC received CPT-11 60 mg $m^{-2}$ intravenously (IV) on days 1, 8 and 15, and cisplatin 80 mg $m^{-2}$ (IV) on day 1 every 4 weeks. Assessments were made of response, survival and toxicities. Sixty-nine were eligible, and evaluable for toxicities and survival, and 64 patients evaluable for response. Thirty-three patients (52%; 95% confidence interval 39–64%) achieved an objective response, with one complete response (2%) and 32 partial responses (50%). The median duration of response was 19 weeks and the overall median survival time was 44 weeks. The 1-year survival rate was 33%. The major toxic effects were leucopenia and diarrhoea. Grade 3 or 4 leucopenia, neutropenia, and diarrhoea occurred in 32 patients (46%), 53 patients (80%), and 13 patients (19%) respectively. A combination of CPT-11 and cisplatin is very effective against non-small-cell lung cancer with acceptable toxicities.

**Keywords:** irinotecan; cisplatin; non-small-cell lung cancer; phase II trial

Lung cancer is a major health care problem throughout the world. Non-small-cell lung cancer (NSCLC) accounts for approximately 75% of all lung cancers. Only a small proportion of patients will be cured by surgery, and most will have metastatic disease and require systemic treatment. Unfortunately, NSCLC is in the group of neoplastic diseases that are relatively chemoresistant. Although a modest survival benefit was noted by meta-analysis (Grilli et al, 1993; Souquet et al, 1993), no regimen is completely effective and none has led conclusively to a cure, and there is no standard chemotherapy programme for this disease (Ginsberg et al, 1993). Therefore, it is imperative to develop combination regimens of new active compounds with novel mechanisms of actions.

Irinotecan (CPT-11) is a water-soluble camptothecin derivative that targets DNA topoisomerase I. CPT-11 has shown a strong anti-tumour activity as a single agent against a broad spectrum of experimental tumours (Kunimoto et al, 1987; Matsuzaki et al, 1988) as well as against various human malignancies including lung cancer (Ohno et al, 1990; Negoro et al, 1991*b*; Tacheuchi et al, 1991; Fukuoka et al, 1992; Masuda et al, 1992*a*; Shimada et al, 1993).

The response rate of 32% to CPT-11 for NSCLC is very encouraging (Fukuoka et al, 1992). Both CPT-11 and cisplatin are active against NSCLC. Because of the differences in mechanisms of action (Andoh et al, 1987; Zwelling and Kohn, 1979) and toxicity profiles (Gottlieb and Drewinko, 1975; Negoro et al, 1991*a*), and with limited cross-resistance (Tsuruo et al, 1988; Noda et al, 1991; Masuda et al, 1992*a*) between the two drugs, the synergism between CPT-11 and its major active metabolite, 7-ethyl-10-hydroxycamptothecin (SN-38) in combination with cisplatin (Kudoh et al, 1993) seems to have enormous clinical potential. Based on these reports, we conducted a phase I trial of the combination of CPT-11 (escalating doses) with cisplatin in advanced NSCLC (Masuda et al, 1992*b*). A response rate of 54% in previously untreated NSCLC patients was very encouraging, even although some patients received relatively low doses of CPT-11. The results of the pilot study led to the multi-institutional phase II study reported here, which was designed to determine the anti-tumour activity and toxicities of a combination of CPT-11 and cisplatin in previously untreated patients with advanced NSCLC.

*Received 12 March 1997*
*Revised 22 July 1997*
*Accepted 21 August 1997*

*Correspondence to:* Noriyuki Masuda, Department of Internal Medicine, Osaka Prefectural Habikino Hospital, 3-7-1 Habikino, Habikino Osaka 583, Japan

### PATIENTS AND METHODS

### Patient selection

Before participation in the study, each patient was examined to make sure he or she met the following criteria: (a) histological diagnosis of non-small-cell lung cancer; (b) stage IIIB or IV disease; (c) no prior chemotherapy or radiotherapy; (d) life expectancy of at least 8 weeks; (e) age ≤ 75 years; (f) performance status of 2 or better on the Eastern Cooperative Oncology Group (ECOG) scale; (g) adequate bone marrow function (leucocyte count ≥ 4000 $\mu l^{-1}$, platelet count ≥ 100 000 $\mu l^{-1}$ and haemoglobin concentration ≥ 9 g $dl^{-1}$), hepatic function (bilirubin ≤ 1.5 mg $dl^{-1}$, transaminases ≤ 2 × upper limit of normal), and renal function (creatinine ≤ upper limit of normal, creatinine clearance ≥ 60 ml $min^{-1}$); (h) free of any concurrent active malignancy; (i) no medical problems sufficiently severe to prevent compliance with the study requirements; and (j) written informed consent of the

**Table 1** Characteristics of eligible patients

| | |
|---|---|
| Total number of patients | 69 |
| Sex | |
| Male | 51 |
| Female | 18 |
| Age: median (range) | 61 years (37–75) |
| Performance status (ECOG): | |
| 0 | 18 |
| 1 | 39 |
| 2 | 12 |
| Stage: | |
| IIIB | 26 |
| IV | 43 |
| Histology | |
| Adenocarcinoma | 51 |
| Squamous cell carcinoma | 15 |
| Large-cell carcinoma | 3 |

patient. Patients were not eligible if they showed allergic reactions to skin-prick test with CPT-11. Pregnant women were also ineligible.

### Treatment plan

CPT-11 at a dose of 60 mg m$^{-2}$ was given in 500 ml of normal saline as a 90 min intravenous infusion on days 1, 8 and 15 every 4 weeks. CPT-11 was provided by Daiichi Pharmaceutical, Tokyo, Japan, and Yakult Honsha, Tokyo, Japan. A 80 mg m$^{-2}$ sample of cisplatin was given on day 1 after the CPT-11 administration every 4 weeks. These doses were the recommended doses of a combination of CPT-11 and cisplatin in a previous phase I/II study (Masuda et al, 1992*b*). During the course of the treatment, the dose of CPT-11 was withheld in instances of leucocyte count <3000 μl$^{-1}$, platelet count <100 000 μl$^{-1}$ or grade ≥2 diarrhoea on the day when the dose was due. Patients with obvious evidence of disease progression or unmanageable toxicity were removed from the study. Patients who were stabilized received at least a second course of treatment; those with objective tumour response were eligible to continue therapy for a maximum of six courses. Before the next course was started, the WBC count had to be 4000 μl$^{-1}$ or higher, the platelet count had to be 100 000 μl$^{-1}$ or higher, the serum creatinine level had to be normal and diarrhoea should be recovered completely.

Subsequent doses were modified on the basis of haematological and non-haematological toxicities. If during the previous cycle the leucocyte nadir was <2000 μl$^{-1}$ and/or the platelet nadir was <50 000 μl$^{-1}$ or if the subsequent cycle was delayed for more than 2 weeks, the dose of CPT-11 was reduced to 50 mg m$^{-2}$. The dose of CPT-11 was also reduced to 50 mg m$^{-2}$ in cases of diarrhoea ≥ grade 3. A prophylactic anti-diarrhoeal regimen was not used in this trial. If the serum creatinine concentration was ≥ 1.7 mg dl$^{-1}$, or the creatinine clearance was below 60 ml min$^{-1}$, the cisplatin dose was reduced to 60 mg m$^{-2}$. Dose adjustments were also made for other toxicities ≥ grade 3, except for nausea and vomiting, and alopecia.

### Evaluation

Patients were evaluated to determine the stage of disease by complete medical history and physical examination, routine chest radiograph, whole-lung tomography, bone scintiscan, computerized tomography of the head, chest and abdomen, and fibreoptic bronchoscopy. The staging procedures were those of the tumour–node–metastasis system (Mountain, 1986). Before the first course, each patient was subject to a complete blood count (CBC), including a differential count and a platelet count, and serum chemistry was used to check renal and hepatic functions, electrolytes and urinalysis. CBC, serum chemistry, electrolytes, urinalysis, and chest radiographs were assessed at least once a week after the initial evaluation. Other appropriate investigations were repeated weekly to evaluate the sites of marker lesions. After the completion of chemotherapy, each patient was restaged with all the tests used during the initial work-up, except for the fibreoptic bronchoscopy. The eligibility, evaluability and response of each patient were assessed by extramural reviewers. The tumour responses and toxicities, except diarrhoea, were classified in accordance with the World Health Organization (WHO) criteria

**Table 2** Response to a combination of CPT-11 and cisplatin treatment

| | Number of patients | CR | PR | NC | PD | NE | Response rate (%) | *P*-value |
|---|---|---|---|---|---|---|---|---|
| Overall | 69 | 1 | 32 | 28 | 3 | 5 | 52* | |
| Sex | | | | | | | | |
| Male | 51 | 1 | 20 | 25 | 2 | 3 | 44 | 0.178 |
| Female | 18 | 0 | 12 | 3 | 1 | 2 | 75 | |
| Performance status (ECOG) | | | | | | | | |
| 0–1 | 57 | 1 | 27 | 24 | 2 | 3 | 52 | 0.866 |
| 2 | 12 | 0 | 5 | 4 | 1 | 2 | 50 | |
| Stage | | | | | | | | |
| IIIB | 26 | 1 | 10 | 12 | 0 | 3 | 48 | 0.116 |
| IV | 43 | 0 | 22 | 16 | 3 | 2 | 14 | |
| Histology | | | | | | | | |
| Adenocarcinoma | 51 | 1 | 26 | 18 | 2 | 4 | 57 | 0.618 |
| Squamous cell cancer | 15 | 0 | 5 | 8 | 1 | 1 | 36 | |
| Large cell cancer | 3 | 0 | 1 | 2 | 0 | 0 | 33 | |

CR, complete response; PR, partial response; NC, no change; PD, progressive disease; NE, non-evaluable;* 95% confidence Interval, 39–64%

© Cancer Research Campaign 1998

**Figure 1** Median survival times in all patients with advanced non-small cell lung cancer were 44 weeks, 49 weeks/stage IIIB (——), and 38 weeks/stage IV disease (- - -) respectively ($P = 0.1185$; log-rank test). The 1-, 2- and 3-year actuarial survival rates in patients with stage IIIB disease were 46.2%, 17.9%, and 9.0%, compared with 25.6%, 4.7%, and 4.7%, respectively, in the patients with stage IV disease

(World Health Organization, 1979). ECOG common toxicity criteria were used to grade diarrhoea. Patients were considered evaluable if they had completed at least one treatment cycle. The duration of each response was defined as the number of days from the start of treatment to its progression. The duration of survival was determined as the number of days from the start of treatment to death or the last follow-up.

### Statistical methods

The method of Kaplan and Meier (1958) was used to derive the survival curve and response duration, and was compared using the log-rank test. Other statistical analyses were performed using the chi-squared test or Fisher's exact test, and $P < 0.05$ was considered to indicate statistical significance. The primary end point was the response rate, which determined sample size. The statistical focus centred on distinguishing a response rate of 60% from the usual expected response rate of 35% obtained with a combination of cisplatin and vindesine in patients with advanced NSCLC. Our design had a power in excess of 80% and less than 5% type I error. Assuming an inevaluability rate of less than 20%, we projected an accrual of ≥ 60 patients.

### RESULTS

Between February 1992 and August 1992, 70 patients participated in the trial. One patient was deemed ineligible because he had stage IIIA disease. The characteristics of the eligible patient population are listed in Table 1. Eighteen patients were women and 51 were men, and the median age was 61 years (range 37–75 years). A total of 26 (38%) patients had stage IIIB disease and 43 (62%) had stage IV disease.

### Response and survival

Sixty-four patients were fully assessable for response. Of the five non-assessable patients, two died of toxicity during the first cycle of treatment, two received treatment only on day 1 because of delayed recovery from leucopenia and paralytic ileus, respectively, and one refused further protocol treatment after day 1 treatment because of severe nausea and vomiting, loss of appetite and general fatigue. Among the 64 assessable patients, one patient achieved a complete response and 32 (50%) had a partial response for an overall response rate of 52% (95% confidence interval, 39%–64%) (Table 2). The median time required to reach remission was 27 days (range 7–63 days). Twenty-eight patients showed no change and three had disease progression. The response rates for adenocarcinoma, squamous cell carcinoma and large-cell carcinoma were 57% (27 out of 47 patients), 36% (5 out of 14 patients) and 33% (one out of three patients) respectively ($P = 0.618$). There was no significant difference in overall response rate when analysed by sex, performance status (0–1 vs 2) ($P = 0.866$), or by stage (stage IIIB vs stage IV) ($P = 0.116$). The median duration of response for all responding patients was 19 weeks (range 8–52 weeks), and that for patients with stage IV disease was 20 weeks (range 8–52 weeks). Of 26 patients with stage IIIB disease, consolidation chest irradiation was used in five patients after reaching a maximal response. An additional six patients with stage IIIB received thoracic radiotherapy after confirming that their responses showed no change.

Of the 69 patients, only two patients (3%) were still alive as of 13 November 1996. One patient was lost to follow-up 74 weeks after the beginning of treatment. The median survival time for all 69 patients was 44 weeks (stage IIIB patients, 49 weeks; stage IV patients, 38 weeks) (Figure 1). The 1-, 2- and 3-year actuarial survival rates in patients with stage IIIB disease were 46.2%, 17.9%, and 9.0%, compared with 25.6%, 4.7% and 4.7%, respectively, in the patients with stage IV disease. The 1-year survival rate was 33.3%, with a 95% confidence interval of 22–44%.

### Toxicity

There were two treatment-related deaths on days 8 and 15 of cycle 1 as a result of neutropenic sepsis coincidentally associated with paralytic ileus. Another nine patients were taken off the study after the first cycle of treatment because of progressive disease (two), patient refusal (one), severe diarrhoea (two), paralytic ileus (one), delayed recovery from leucopenia (one), pulmonary toxicity (one) and skin rash (one). Fifty-eight patients received multiple courses of treatment in successive cycles. A total of 175 courses were given; all were valid for toxicity analysis (mean cycles per patient, 2.5: range 1–6). Reduction of the cisplatin dose was required in only two patients (three cycles). Details of the CPT-11 dose actually delivered are listed in Table 3. Thirty-six (52%) of 69 patients could receive CPT-11 treatment three times during cycle 1. Virtually all episodes of severe leucopenia and/or diarrhoea were observed during cycle 1 as dose modifications were made in subsequent cycles. Consequently, it was possible to deliver the full doses of CPT-11 treatment (three times) in 59% of the entire 175 cycles, suggesting little evidence of cumulative toxicity during the subsequent courses of treatment. The reasons why patients could not receive CPT-11 treatment three times in the first course were treatment-induced leucopenia (33%), thrombocytopenia (9%) and/or diarrhoea (17%).

Table 4 shows the maximum toxicities experienced during the treatment. The most frequent toxicity was myelosuppression, which primarily affected leucocytes: grade 3 or 4 neutropenia occurred in 23% and 9% of patients respectively. The leucocyte nadir usually occurred around day 17, with recovery in most patients by day 29. Little cumulative toxicity was detected in the subsequent courses. Of 66 patients, 28 (42%) developed grade 3 neutropenia (absolute neutrophil count = 500 to 1000 $\mu l^{-1}$) and 25 (38%) had grade 4

© Cancer Research Campaign 1998

Table 3 Number of CPT-11 doses actually delivered to patients

| | During the first course Number of patients (%) ($n$ = 69) | During all courses Numbers of courses (%) ($n$ = 175) |
|---|---|---|
| Full dose (days 1, 8, 15) | 36 (52) | 104 (59) |
| Twice | | |
| (days 1, 8) | 23 (33) | 42 (24) |
| (days 1, 15) | 5 (7) | 17 (10) |
| Only once (day 1) | 5 (7) | 12 (7) |

Table 4 Toxicities of CPT-11/cisplatin combination chemotherapy

| Toxicity (%) | Number of patients assessable | WHO grade | | | | Number of ≥ grade 3 |
|---|---|---|---|---|---|---|
| | | 1 | 2 | 3 | 4 | |
| Leucopenia | 69 | 8 | 24 | 23 | 9 | 32 (46) |
| Neutropenia | 66 | 1 | 8 | 28 | 25 | 53 (80) |
| Thrombocytopenia | 69 | 11 | 2 | 2 | 4 | 6 (9) |
| Anaemia | 68 | 15 | 19 | 20 | 4 | 24 (35) |
| Nausea/vomiting | 69 | 18 | 21 | 24 | – | 24 (35) |
| Diarrhoea | 69 | 26 | 16 | 10 | 3 | 13 (19) |
| Alopecia | 69 | 21 | 30 | 5 | – | 5 (7) |
| Pulmonary toxicity | 69 | 0 | 0 | 0 | 1 | 1 (1) |
| Ileus | 69 | 0 | 0 | 0 | 3 | 3 (4) |
| Fever | 69 | 1 | 5 | 0 | 0 | 0 (0) |
| Elevation of serum creatinine | 69 | 8 | 1 | 0 | 0 | 0 (0) |
| Elevation of transaminases | 68 | 12 | 1 | 0 | 0 | 0 (0) |
| Mucositis | 69 | 2 | 0 | 0 | 0 | 0 (0) |

neutropenia (absolute neutrophil count < 500 µl). Among 69 patients, 29 (42%) patients received granulocyte colony-stimulating factor when they experienced leucopenia. Transient eosinophilia in 10% or more of leucocytes was observed in 12 (18%) of 67 patients. Thrombocytopenia remained infrequent throughout the study: grade 3 and 4 toxicity only occurred in 3% and 6% of the patients respectively. Thirty-five per cent of patients had grade 3 or 4 anaemia.

Non-haematological toxicities were also significant. Gastrointestinal toxicity was a prominent toxic effect of this treatment. Grade 3 nausea and vomiting occurred in 24 (35%) patients. Grade 3 and 4 diarrhoea was observed in 10 (14%) and 3 (4%) patients. Maximal grade diarrhoea occurred on a median of day 11 (range: day 1–18), and recovery was observed in a median of 4 days (range: 1–12 days). Although treatment of diarrhoea was left at the discretion of the treating physicians, most of the patients with diarrhoea were treated with loperamide, as reported by Abigerges et al (1994). However, three (4%) patients experienced grade 4 paralytic ileus, two of whom died early in the treatment as described above. No severe toxicities were observed in the urinary bladder, kidney or liver. Grade 4 pulmonary toxicity occurred only in one (1%) patient, who developed severe hypoxaemia during his first course of treatment and required mechanical ventilation.

### DISCUSSION

As the majority of patients die from advanced NSCLC, there is a compelling need for more effective treatment that offers a realistic possibility of improving the survival in these patients. The current multi-institutional phase II trial of CPT-11 and cisplatin in combination for advanced NSCLC demonstrates the encouraging response rate of 52%, confirming the preliminary response rate of 54% obtained in a previous phase I trial in advanced NSCLC (Masuda et al, 1992*b*). A response rate of 30–40%, a median survival time of 6–9 months, and a 1-year survival of approximately 20% are commonly seen in treatments with cisplatin-based regimens, usually in combination with etoposide or a vinca alkaloid (Cellerino et al, 1990; Splinter, 1991; Bunn, 1992). Therefore, the response rate obtained in this study is in the upper range of the reported trials in NSCLC among combination chemotherapy regimens currently in use. As the high response rate of 52% and the 1-year survival rate of 33% obtained here are encouraging, this combination holds promise not only as primary therapy for patients with metastatic disease (stage IV) but also for patients with more localized disease in the adjuvant or neoadjuvant (induction) setting in combination with surgery and/or radiotherapy. In the last two instances a better efficacy of this combination regimen is expected because improved response rates to chemotherapy in patients with a lower tumour burden have been demonstrated in patients with NSCLC (Donnadieu et al, 1991) and other solid tumours (Hong and Bromer, 1983). Recently, a number of other new chemotherapeutic agents with substantial activity against NSCLC, including vinorelbine, paclitaxel, docetaxel and gemicitabine, have become available (Lilenbaum and Green, 1993). The inclusion of these agents in combination chemotherapy regimens may provide a substantial improvement in the treatment of this disease. Pirker et al (1995) reported that paclitaxel and cisplatin combination showed a response rate of 35% in patients with stage IIIB or IV NSCLC. Langer et al (1995) observed a very high response rate of 62%, a median survival time of 53 weeks, and a 1-year survival of 54% with a combination of paclitaxel and

 © Cancer Research Campaign 1998

carboplatin in 53 patients with advanced NSCLC. In contrast, Johnson et al (1996) used the same paclitaxel and carboplatin combination and obtained a relatively disappointing response rate of 27%, a median survival time of 38 weeks, and a 1-year survival of 32% in patients with stage IV NSCLC. Prospective randomized trials that compare these new combination regimens with an existing cisplatin-containing regimen are necessary to determine whether or not these new agents will truly improve therapeutic options for patients with advanced NSCLC.

One of the major arguments against the use of chemotherapy for NSCLC concerns the considerable toxicity. As expected, leucopenia and diarrhoea, which are typical toxicities of CPT-11 (Fukuoka et al, 1992), were the major toxicities of this combination regimen, with the most severe toxicities occurring during cycle 1 (Table 3). The marked interpatient variation in the toxicities, which is a well-known feature of CPT-11 (Masuda et al, 1992*b*; 1993), was also noted in this trial. Two patients died on days 8 and 15 after experiencing severe leucopenia simultaneously with severe diarrhoea. These lethal toxicities occurred very rapidly with unpredictable severity during cycle 1. Patients who could tolerate cycle 1 without major toxic effects were less likely to experience severe toxicities in subsequent cycles without dose adjustments. Despite the dose modification procedure, in which CPT-11 was withheld if the leucocyte count was < 3000 $\mu l^{-1}$ when treatment was due, grade 3 or 4 leucopenia more frequently occurred in this trial than in a previous phase II trial of CPT-11 given as a single agent (46% vs 25%) (Fukuoka et al, 1992), suggesting at least an additive leucopenic effect in combination with cisplatin. With the availability of recombinant human granulocyte colony-stimulating factor (rhG-CSF), it has become possible to reduce the severity and duration of leucopenia induced by cytotoxic chemotherapy (Gabrilove et al, 1988). At the time of the current trial rhG-CSF had already become commercially available. However, the frequency of severe leucopenia did not decrease in the current study. This may be in part explained by the fact that the use of rhG-CSF is allowed in Japan only in patients who experience leucopenia of grade 3 or worse. Diarrhoea was another principal dose-limiting toxicity observed with this combination regimen (Table 4). The recent introduction of the use of high-dose loperamide by Abigerges et al (1994) has substantially reduced the previously marked CPT-11-induced diarrhoea. However, 19% of patients still suffered from grade 3 or 4 delayed diarrhoea in this trial. Diarrhoea of grade 3 or 4 occurring coincidentally with grade 4 leucopenia and high fever was especially life-threatening or lethal, in spite of active supportive care including vigorous intravenous hydration and intravenous administration of broad-spectrum antibiotics. Much improvement in the control of this toxic effect is required to make it more easily to apply safely to a wide variety of patients. Therefore, further studies to pursue effective methods of overcoming this late diarrhoea are necessary.

In conclusion, the combination of CPT-11 and cisplatin is an effective regimen in advanced NSCLC, with a response rate and survival time that favourably compare with the results of many other combination chemotherapy regimens in advanced NSCLC. As the combination of cisplatin and vindesine is the regimen that has been reported to prolong survival by 10 weeks when compared with the best supportive care (Grilli et al, 1993; Souquet et al, 1993; Non-small Cell Lung Cancer Collaborative Group, 1995), it seems useful to compare the effect of these two combination chemotherapy regimens. Its final role in the treatment of this disease will be made clear by a currently on-going prospective randomized trial comparing CPT-11 alone vs a combination of CPT-11 and cisplatin vs a combination of cisplatin and vindesine in treating patients with advanced disease. Another trial comparing a combination of CPT-11 and cisplatin vs a combination of cisplatin and vindesine in advanced non-small cell lung cancer is also in progress in Japan.

### ACKNOWLEDGEMENTS

We wish to thank Mrs Kyoji Tamanoi and Fumiyasu Fukuda for their help in the data collection and analysis. This work was supported by a grant from Daiichi Pharmaceutical (Tokyo, Japan) and Yakult Honsha (Tokyo, Japan)

### REFERENCES

Abigerges D, Armand JP, Chabot GG, Da Costa L, Fadel E, Cote C, Herait P and Gandia D (1994) Irinotecan (CPT-11) high-dose escalation using intensive high-dose loperamide to control diarrhoea. *J Natl Cancer Inst* **86**: 446–449

Andoh T, Ishii K, Suzuki Y, Ikegami Y, Kusunoki Y, Takemoto Y and Okada K (1987) Characterization of a mammalian mutant with a camptothecin-resistant DNA topoisomerase I. *Proc Natl Acad Sci USA* **84**: 5565–5569

Bunn PA (1992) The expanding role of cisplatin in the treatment of non-small cell lung cancer? *Semin Oncol* **16**: 10–21

Cellerino R, Tummarello D and Piga A (1990) Chemotherapy or not in advanced non-small cell lung cancer? *Lung Cancer* **6**: 99–109

Donnadieu N, Paesmans M and Sculier JP (1991) Chemotherapy of non-small-cell lung cancer according to disease extent: a meta-analysis of the literature. *Lung Cancer* **7**: 243–253

Fukuoka M, Niitani H, Suzuki A, Motomiya M, Hasegawa K, Nishiwaki Y, Kuriyama T, Ariyoshi Y, Negoro S, Masuda N, Nakajima S and Taguchi T (1992) A phase II study of CPT-11, a new derivative of camptothecin, for previously untreated non-small-cell lung cancer. *J Clin Oncol* **10**: 16–20

Gabrilove JL, Jakubowski A, Scher H, Sternberg C, Wong G, Grous J, Yagoda A, Fain K, Moore MAS, Clarkson B, Oettgen HF, Alton K, Welte K and Souza L (1988) Effect of granulocyte colony-stimulating factor on neutropenia and associated morbidity due to chemotherapy for transitional-cell carcinoma of the urothelium. *N Engl J Med* **318**: 1414–1422

Ginsberg RJ, Kris MG and Armstrong JG (1993) Cancer of the lung. In *Cancer: Principle and Practice of Oncology*, Devita VT, Hellman S and Rosenberg SA (eds), pp. 673–723. Lippincott: Philadelphia

Gottlieb J and Drewinko B (1975) Review of the current clinical status of platinum coordination complexes in cancer chemotherapy. *Cancer Chemother Rep* **59**: 621–628

Grilli R, Oxman A and Julian JA (1993) Chemotherapy for advanced non-small-cell lung cancer. How much benefit is enough? *J Clin Oncol* **11**: 1866–1872

Hong WK and Bromer R (1983) Chemotherapy in head and neck cancer. *N Engl J Med* **308**: 75–59.

Johnson DH, Paul DM, Hande KR, Shyr Y, Blanke C, Murphy B, Lewis M and DeVore III RF (1996) Paclitaxel plus carboplatin in advanced non-small-cell lung cancer: a phase II trial. *J Clin Oncol* 2054–2060

Kaplan E and Meier P (1958) Nonparametric estimation from incomplete observations. *J Am Stat Assoc* **53**: 457–481

Kudoh S, Takada M, Masuda N, Nakagawa K, Itoh K, Kusunoki Y, Negoro S, Matsui K, Takifuji N, Morino H and Fukuoka M (1993) Enhanced antitumor efficacy of a combination of CPT-11, a new derivative of camptothecin, and cisplatin against human lung tumor xenografts. *Jpn J Cancer Res* **84**: 203–207

Kunimoto T, Nitta K, Tanaka T, Uehara N, Baba H, Takeuchi M, Yokokura T, Sawada S, Miyasaka T and Mutai M (1987) Antitumour activity of 7-ethyl-10-[4-(1-piperidino)-1-piperidino]carbonyloxy-camptothecin, a novel water-soluble derivative of camptothecin, against murine tumours. *Cancer Res* **47**: 5944–5947

Langer CJ, Leighton JC, Comis RL, O'Dwyer PJ, McAleer CA, Bonjo CA, Engstrom PF, Litwin S and Ozols RF (1995) Paclitaxel and carboplatin in combination in the treatment of advanced non-small-cell lung cancer: a phase II toxicity, response, and survival analysis. *J Clin Oncol* **13**: 1860–1870

Lilenbaum R and Green M (1993) Novel chemotherapeutic agents in the treatment of non-small-cell lung cancer. *J Clin Oncol* **11**: 1391–1402

© Cancer Research Campaign 1998

Masuda N, Fukuoka M, Kusunoki Y, Matsui K, Takifuji N, Kudoh S, Negoro S, Nishioka M, Nakagawa K and Takada M (1992*a*) CPT-11: a new derivative of camptothecin for the treatment of refractory or relapsed small-cell lung cancer. *J Clin Oncol* **10**: 1225–1229

Masuda N, Fukuoka M, Takada M, Kusunoki Y, Negoro S, Matsui K, Kudoh S, Takifuji N, Nakagawa K and Kishimoto S (1992*b*) CPT-11 in combination with cisplatin for advanced non-small-cell lung cancer. *J Clin Oncol* **10**: 1775–1780

Masuda N, Fukuoka M, Kudoh S, Kusunoki Y, Matsui K, Takifuji N, Nakagawa K, Tamanoi M, Nitta T, Hirashima T, Negoro S and Takada M (1993) Phase I and pharmacologic study of irinotecan in combination with cisplatin for advanced lung cancer. *Br J Cancer* **68**: 777–782

Matsuzaki T, Yokokura T, Mutai M and Tsuruo T (1988) Inhibition of spontaneous and experimental metastasis by a new derivative of camptothecin, CPT-11, in mice. *Cancer Chemother Pharmacol* **21**: 308–312

Mountain CF (1986) A new international staging system for lung cancer. *Chest* **89**: 225S–233S

Negoro S, Fukuoka M, Masuda N, Takada M, Kusunoki Y, Matsui K, Takifuji N, Kudoh S, Niitani H and Taguchi T (1991*a*) Phase I study of weekly intravenous infusions of CPT-11, a new derivative of camptothecin, in the treatment of advanced non-small-cell lung cancer. *J Natl Cancer Inst* **83**:1164–1168

Negoro S, Fukuoka M, Niitani H and Taguchi T (1991*b*) Phase II study of CPT-11, new camptothecin derivative, in small cell lung cancer (SCLC). *Proc Am Soc Clin Oncol* **10**: 241

Noda K, Takeuchi S, Yakushiji M and CPT-11 study group on gynecologic Malignancy iJ (1991) Late phase II study of CPT-11, new camptothecin derivative, in cervical and ovarian carcinoma. *Proc World Cong Gynecol Obstet* **13**: 279

Non-small Cell Lung Cancer Collaborative Group (1995) Chemotherapy in non-small cell lung cancer: a meta-analysis using updated data on individual patients from 52 randomised clinical trials. *Br Med J* **311**: 899–909

Ohno R, Okada K, Masaoka T, Kuramoto A, Arima T, Yoshida Y, Ariyoshi H, Ichimaru M, Sakai Y, Oguro M, Ito Y, Morishima Y, Yokomaku S and Ota K (1990) An early phase II study of CPT-11: a new derivative of camptothecin, for the treatment of leukemia and lymphoma. *J Clin Oncol* **8**: 1907–1912

Pirker R, Krajnik G, Zöchbauer S, Malayeri R, Kneussl M and Huber H (1995) Paclitaxel/cisplatin in advanced non-small-cell lung cancer (NSCLC). *Ann Oncol* **6**: 833–835

Shimada Y, Yoshino M, Wakui A, Nakao I, Futatsuki K, Sakata Y, Kambe M and Taguchi T (1993) Phase II study of CPT-11, a new camptothecin derivative, in metastatic colorectal cancer. *J Clin Oncol* **11**: 909–913

Souquet PJ, Boissel JP, Cellerino R, Cormier Y, Ganz PA, Kaasa S, Pater JL, Qunoix E, Rapp E, Tumarello D, Williams J, Woods Bl and Bernard JP (1993) Polychemotherapy in advanced non small cell lung cancer: a meta-analysis. *Lancet* **342**: 19–21

Splinter TAW (1991) Response rate as criterion to evaluate chemotherapy in non-small cell lung cancer. *Lung Cancer* **7**: 91–104

Tacheuchi S, Dobashi K, Fujimoto S, Tanaka K, Suzuki M, Terashima Y, Hasumi K, Akiya K, Negishi Y, Tamaya T, Tanizawa O, Sugawa T, Umesaki N, Sekiba T, Aono T, Nakano H, Noda K, Shiota M, Yakushiji M, Sugiyama T, Hashimoto M, Yamaji A, Takamizawa H, Sonoda T, Takeda Y, Tomoda Y, Ohta M, Ozaki M, Hirabayashi K, Hiura M, Hatae M, Nishigaki K and Taguchi T (1991) A late phase II study of CPT-11 on uterine cervical cancer and ovarian cancer. *Gan To Kagakuryoho* **18**: 1681–1689

Tsuruo T, Matsuzaki T, Matsushita M, Saito H and Yokokura T (1988) Antitumour effect of CPT-11, a new derivative of camptothecin, against pleiotropic drug-resistant tumours *in vitro* and *in vivo*. *Cancer Chemother Pharmacol* **21**: 71–74

World Health Organization (1979) *WHO Handbook for Reporting Results of Cancer Treatment. WHO Offset Publication No. 48*. World Health Organization: Geneva

Zwelling L and Kohn K (1979) Mechanism of action of cisdichlorodiammineplatinum (II). *Cancer Treat Rep* **63**: 1439–1444

### APPENDIX

The following doctors participated in this study: Dr Mitsuo Asakawa, Sapporo Medical University, Hokkaido; Dr Tetsuo Shimizu, National Dohoku Hospital, Hokkaido; Dr Yuji Inoue, Asahikawa Red-Cross Hospital, Hokkaido; Dr Hiroyuki Nakai, The Research Institute for Tuberculosis and Cancer, Tohoku University, Miyagi; Dr Ryusei Saitoh, National Nishi-gunma Hospital, Gunma; Dr Yoshikuni Saito, Tochigi Cancer Center, Tochigi; Drs Takayuki Kuriyama, Institute of Pulmonary Cancer Research, Chiba University; Dr Senri Hirakawa, Gifu University School of Medicine, Gifu; Dr Kohji Nakahara, Gifu Municipal Hospital, Gifu; Drs Yutaka Ariyoshi and Takahiko Sugiura, Aichi Cancer Center, Aichi; Dr Harumichi Ikegami, The Center for Adult Diseases, Osaka; Drs Shigenori Nakajima and Yuji Tohda, Kinki University School of Medicine, Osaka; Dr Shinei Ryu, Shimonoseki Kosei General Hospital, Yamaguchi; Dr Takeshi Ogura, Tokushima University School of Medicine, Tokushima; Dr Jiro Takahara, Kagawa Medical School, Kagawa; Dr Tadashi Kamei, Kagawa Prefectual Central Hospital, Kagawa; Dr Nobuyuki Hara, National Kyushu Cancer Center, Fukuoka; Dr Masamoto Nakano, Nagasaki City Hospital, Nagasaki; Dr Jun Araki, Sasebo General Hospital, Nagasaki; Dr Shinichi Hayasaka, Kumamoto Central Hospital, Kumamoto.

© Cancer Research Campaign 1998